# Long-Term Impact of Social Isolation and Molecular Underpinnings

**DOI:** 10.3389/fgene.2020.589621

**Published:** 2020-10-22

**Authors:** Rodrigo G. Arzate-Mejía, Zuzanna Lottenbach, Vincent Schindler, Ali Jawaid, Isabelle M. Mansuy

**Affiliations:** ^1^Laboratory of Neuroepigenetics, Medical Faculty of the University of Zurich and Department of Health Science and Technology of the Swiss Federal Institute of Technology, Neuroscience Center Zurich, Zurich, Switzerland; ^2^Swiss Federal Institute of Technology Zurich, Zurich, Switzerland

**Keywords:** non-coding RNAs, microRNA, long non-coding (lnc) RNAs, epigenetics, social isolation, behavior, COVID-19

## Abstract

Prolonged periods of social isolation can have detrimental effects on the physiology and behavior of exposed individuals in humans and animal models. This involves complex molecular mechanisms across tissues in the body which remain partly identified. This review discusses the biology of social isolation and describes the acute and lasting effects of prolonged periods of social isolation with a focus on the molecular events leading to behavioral alterations. We highlight the role of epigenetic mechanisms and non-coding RNA in the control of gene expression as a response to social isolation, and the consequences for behavior. Considering the use of strict quarantine during epidemics, like currently with COVID-19, we provide a cautionary tale on the indiscriminate implementation of such form of social isolation and its potential damaging and lasting effects in mental health.

## Introduction

Social behavior is a major life component of many organisms. Proper behavior in response to environmental conditions and signals is critical for development, reproduction, and survival (Chen and Hong, 2018). In mammals, social behavior is exquisitely regulated by brain mechanisms that depend on the control of gene expression during development and in response to life experiences (Cole et al., 2007; Zayed and Robinson, 2012; Chen and Hong, 2018). Accumulating evidence suggests that chromatin-based processes and molecular mechanisms including DNA methylation, non-coding RNA (ncRNA) and transcription factors play critical roles in the control of gene regulatory networks that establish and modulate social behavior (Yao et al., 2016; Hwang et al., 2017; Bludau et al., 2019; Seebacher and Krause, 2019; Nord and West, 2020). However today, how the modulation of gene expression can shape behavioral responses to experiences, such as social isolation, during early postnatal development and in adult life is poorly understood (Hilakivi et al., 1989; Weiss et al., 2004; Zelikowsky et al., 2018). Particularly, when social interactions are perturbed by social isolation, a special condition during periods of pandemics like the one we are currently going through, this can directly impact mental health and have consequences throughout life.

This review provides a comprehensive overview of the effects of prolonged periods of social isolation on the body and describes the known molecular events leading to behavioral alterations. We review the current evidence linking social isolation with changes in gene expression in the brain, and the effects on regulators of genome activity such as epigenetic modifiers, ncRNA and transcription factors. Direct functional evidence supporting the role of miRNAs and long ncRNAs (lncRNAs) as modulators of social behavior and their link to behavioral abnormalities observed during and after prolonged social isolation are discussed. Finally, we reflect on the effects that prolonged social isolation, such as observed during strict quarantine in epidemics, can have on mental health and discuss interventions that may help to ameliorate their burden.

### Effects of Social Isolation in Humans

In humans, chronic social isolation can have detrimental health effects (House et al., 1988) (summarized in Table 1). Social isolation is associated with increased blood pressure, C-reactive protein, and fibrinogen levels (Shankar et al., 2011). It is also associated with an increased risk to be inactive (Shankar et al., 2011; Schrempft et al., 2019), have motor decline (Buchman et al., 2010) and impaired cognitive functions (Shankar et al., 2013). Loneliness or living alone has been linked with poorer immediate and delayed recall (Shankar et al., 2013) and dementia (Holwerda et al., 2014), as well as higher odds of mental health problems (Coyle and Dugan, 2012). Social isolation can as well result in health-risk behaviors, smoking (Shankar et al., 2011), and reduced self-related physical health (Cornwell and Waite, 2009; Coyle and Dugan, 2012). Therefore, social isolation affects physiology, cognition, and behavior in humans.

**TABLE 1:** Physiological and mental health effects of decreased social interactions in humans.

Effects of non-enforced loneliness and social isolation				
Exposure	Age	Participants (*N*)	Effects of exposure	References
Loneliness Social isolation Old age	50+ Mean: 66.9	8,688	-Social isolation was positively associated with blood pressure, C-reactive protein, and fibrinogen levels. -Social isolation and loneliness were associated with higher risk of being inactive, smoking, as well as reporting multiple health-risk behaviors.	Shankar et al., 2011
Social disconnectedness Perceived isolation Old age	57–85	2910	-The correlation between social disconnectedness and perceived isolation is only weak to moderate in strength (*r* = 0.25, *p* < 0.001). -Results indicate that social disconnectedness and perceived isolation are independently associated with lower levels of self-rated physical health.	Cornwell and Waite, 2009
Social isolation Loneliness Old age	65–84	4004	-The mortality hazard ratio for feelings of loneliness was 1.30 [95% confidence interval (CI) 1.04–1.63] in men and 1.04 (95% CI 0.90–1.24) in women. -No higher risk of mortality was found for social isolation.	Holwerda et al., 2012
Feelings of loneliness (FoL) Living alone Old age	Elderly people mean age: 76.5	3620 community-dwelling elderly people	-Living alone and FoL were both independent predictors of death after 22 years of follow-up (hazard ratio, 1.14; 95% CI, 1.05–1.23; *p* = 0.001) and (hazard ratio, 1.20; 95% CI, 1.08–1.33; *p* =0.001), respectively. -No significant interaction was found between feelings of loneliness and living alone (β = 0.08; relative risk = 0.85; 1.40; *p* = 0.48).	Tabue Teguo et al., 2016
Feelings of loneliness Social isolation	Older persons	2173 non-demented community-living older persons	-Factors positively associated with developing dementia: living alone (*p* = 0.001), no longer being married (*p* = 0.001), feelings of loneliness (*p* = 0.000) and receiving social support (*p* =0.000). -Social isolation was not associated with a higher dementia risk in multivariate analysis.	Holwerda et al., 2014
Loneliness Old age	Older people Average age: 79.67	985 persons without dementia ∼25% male	-The level of loneliness at baseline was associated with the rate of motor decline (Estimate, −0.016; *SE:* 0.006, *p* = 0.005). -When terms for both feeling alone (loneliness) and being alone were considered together in a single model, both were relatively independent predictors of motor decline.	Buchman et al., 2010
Social isolation Loneliness Old age	Older adults	11,825	-Loneliness and social isolation were not highly correlated with one another (*r* = 0.201, *p* = 0.000). -Loneliness was associated with higher odds of having a mental health problem (OR: 1.17; CI: [1.13, 1.21], *p* = 0.000). -Isolation was associated with higher odds of reporting one’s health as being fair/poor (OR: 1.39; CI: [1.21, 1.59], *p* = 0.000).	Coyle and Dugan, 2012
Social isolation Loneliness	Mean age at baseline: 65.6 years	6034	-Baseline isolation was associated with decreases in all cognitive function measures at follow-up (β = −0.05 to −0.03, *p* < 0.001). -Loneliness was associated with poorer immediate recall (β = −0.05, *p* < 0.001) and delayed recall (β = −0.03, *p* = 0.02). -Interaction between educational level and both isolation (*p* = 0.02) and loneliness (*p* = 0.01) for delayed recall, such that isolation and loneliness were associated with poorer recall only among those with low levels of education.	Shankar et al., 2013
Social isolation Loneliness Old age	Aged 50–81 years (mean 66.01)	267 community-based men (*n* = 136) and women (*n* = 131)	-Total 24 h activity counts were lower in isolated compared with non-isolated respondents (β = −0.130, *p* = 0.028). -Loneliness was not associated with physical activity or sedentary behavior.	Schrempft et al., 2019
Transition to living alone, Old age	65+	4587	-Living consistently alone did confer increased odds for caseness. -Living alone in later life is not in itself a strong risk factor for psychological distress. -Greater risk of caseness for women, risk increases with age. -A likelihood ratio test confirms that the key interaction between time and living arrangements adds significant explanatory value to the model (*p* < 0.001).	Stone et al., 2013
Solitude	Mean age: 21	44 female college students	-Cortisol levels were significantly higher when individuals were alone. -Trait affectivity moderated the association between solitude and cortisol.	Matias et al., 2011
**Effects of enforced social isolation**				
30 days isolation	(Age mean: 36.3 ± 7.2) (Age mean: 31.8 ± 8.7)	16 isolated participants 17 non-isolated	30 days of isolation do not have a significant impact on brain activity, neurotrophic factors, cognition, or mood, even though stress levels were significantly increased during isolation.	Weber et al., 2019
Quarantine	64% were 26–45 years of age	129 quarantined persons	-Symptoms of post-traumatic stress disorder (PTSD) and depression were observed in 28.9% and 31.2% of responders. (median duration of quarantine: 10 days). -Longer quarantine was associated with an increased prevalence of PTSD symptoms. -Acquaintance with or direct exposure to someone with a diagnosis of SARS was also associated with PTSD and depressive symptoms.	Hull, 2005
Stress related to epidemic	Mean age: ∼39	Randomly selected employees (*n* = 549) of a hospital in Beijing	-About 10% of the respondents had experienced high levels of post-traumatic stress (PTS) symptoms since the SARS outbreak. -Respondents who had been quarantined, or worked in high-risk locations such as SARS wards, or had friends or close relatives who contracted SARS, were 2 to 3 times more likely to have high PTS symptom levels, than those without this exposure. -Altruistic acceptance of work-related risks was negatively related to PTS levels.	Wu et al., 2009
SARS quarantine	Mean age: 49	1057	-Self-reported compliance with all required quarantine measures was low (15.8 ± 2.3%), although significantly higher when the rationale for quarantine was understood (*p* = 0.018). -Health-care workers (HCW) experienced greater psychological distress, including symptoms of PTSD (*p* < 0.001). -Increasing perceived difficulty with compliance, HCW, longer quarantine and compliance with quarantine requirements were significant contributors to higher IES-R scores.	Reynolds et al., 2008
9 days SARS quarantine	Mean age: 39	338 hospital staff	-Quarantine was detected as a relevant factor leading to acute stress disorder (5%) -Feeling stigmatized and rejected in the neighborhood (20%) -Considered resignation (9%)	Bai et al., 2004
2 weeks after contact with MERS patients	Mean age: 44	1656	-During the isolation period, 7.6% of participants had anxiety symptoms, 16.6% had feelings of anger. -After 4–6 weeks, 3% of participants had anxiety symptoms, 6.4% had feelings of anger. -Risk factors: inadequate supplies, social networking activities, history of psychiatric illnesses, financial loss.	Jeong et al., 2016
SARS quarantine	Mean age: 39	903	-Most residents of the first officially recognized site of community outbreak were affected by stigma. -Forms: being shunned, insulted, marginalized, rejected. -Stigma was associated with psychical distress.	Lee et al., 2005
Hospital staff SARS quarantine	Mean age: ∼40	549 hospital employees, 104 quarantined	-Increased odds of having depression 3 years later: being single, having been quarantined, exposure to other traumatic events before SARS, perceived SARS-related risk level. -Decreased odds: altruistic acceptance of risk.	Liu et al., 2012b
SARS quarantine	Mean age: 44	333 nurses	-Lower levels of avoidance behavior, emotional exhaustion, anger, and burnout: high levels of vigor, organizational support, trust in equipment, low levels of contact with SARS patients, time spent in quarantine.	Marjanovic et al., 2007
10 days SARS quarantine	ND^∗^	99 Health care workers 19 patients with SARS	-Patients with SARS reported fear, loneliness, boredom, anger, and worries about family members, anxiety, insomnia, uncertainty, and stigmatization. -Staff: fear of contagion and infecting family, uncertainty, and stigmatization.	Maunder et al., 2003
City isolation because of SARS	ND^∗^	187	-26.2% of participants had psychological disorders. -Prediction factors: income reduction (odds ratio: 25.0), gender, range of activities, eating restrictions, restrictions in going out, disinfection of clothing, infection control.	Mihashi et al., 2009
Ebola quarantine	ND^∗^	432 (focus group) and 30 (interviews)	-High level of social insecurity. -Stress because of forced cremation of death for poor people. -Quarantine raised condemnation, strengthened stigmatization, created socio-economic distress.	Pellecchia et al., 2015
10 days SARS quarantine	Mean age: 43	10 health-care workers	Experienced stigma, fear, frustration.	Robertson et al., 2004
Equine influenza quarantine	Mean age: ∼40	2760 horse owners	34% reported high psychological distress (12% in the general population).	Taylor et al., 2008
H1N1 quarantine	Mean age: 20	419 undergraduates	No significant differences between quarantined and non-quarantined group.	Wang et al., 2017
MERS quarantine	ND*	6231	1221 people placed in quarantine experienced psychological and emotional difficulties, 350 required continuing services.	Yoon et al., 2016

*ND^∗^ = No Data.*

### Effects of Social Isolation in Animal Models

In rodents, social isolation has multiple effects on physiology and behavior (summarized in Table 2). Chronic social isolation (at least 2 weeks) results in complex behavioral responses characterized by increased aggressive behavior toward a submissive intruder, enhanced reactivity to footshock, and freezing to threatening ultrasonic stimulus (Zelikowsky et al., 2018). It also reduces time spent in the center of the arena during open field test (OFT) and increases the propensity to jump off an elevated plus maze (EPM) test (Zelikowsky et al., 2018). Chronically-isolated rodents spend less time interacting with a novel individual, but more time closer to a predator (Zelikowsky et al., 2018). They also have higher anxiety, depression, and anhedonia-like behaviors (Wallace et al., 2009), indicating that chronic social isolation alters behavioral responses in multiple ways.

**TABLE 2:** Effects of social isolation (SI) in animal models.

Exposure	Organism	Duration of SI	Effects of exposure	References
Social isolation, running, adjusting corticosterone levels	Sprague-Dawley rats:adult, male	12 days	High corticosterone levels in response to stress after social isolation cause running to decrease neurogenesis.	Stranahan et al., 2006
Social isolation Antidepressants (fluoxetine, desipramine) Enriched environment	Swiss mice, adult, male/female	1 week	-Decreased neurogenesis in the olfactory bulb and ventral hippocampus, reduced norepinephrine in OB, and decreased NE and serotonin in the dorsal hippocampus. -Many effects can be prevented by fluoxetine and desipramine.	Guarnieri et al., 2020
Social environments	Male Sprague-Dawley rats (young)	4 or 8 weeks	-Decreased newborn neurons in the dentate gyrus and reduced long-term potentiation in the hippocampus.	Lu et al., 2003
Social isolation	Male Lister Hooded rats; 28 days old	8 weeks	-Volume loss of medial prefrontal cortex, but no loss in neuron number- > loss of volume of the neuropil.	Day-Wilson et al., 2006
Social isolation	Mice: male, 9 weeks old	4 weeks	-Alteration of neuroplasticity related genes.	Ieraci et al., 2016
Exposure to chronic stress (social deprivation)	Mice: male, 3-month-old (C57BL/6)	3 weeks	-Increased HPA axis reactivity and reduced BDNF levels.	Berry et al., 2012
Chronic social isolation stress (CSIS) Acute stress	Rats	21 days of chronic social isolation	Changes in redox-status associated with decreased Hsp70i protein expression enabled NF-jB translocation into the nucleus, causing increased cytosolic nNOS and iNOS protein expression- > oxidative stress.	Zlatković and Filipović, 2013
Social isolation	Rats	30 days	The decrease in neuroactive steroids could be due to a decrease in activity of the HPA axis or the peripheral benzodiazepine receptor response.	Serra et al., 2004
Social isolation	Prairie voles, female/male, adult (2 months old)	4 weeks	Reduction in hypothalamic CRH-R2 and increase in hippocampal CRH-R2 expression	Pournajafi-Nazarloo et al., 2011
Loss of bonded partner, monogamous rodent	Prairie voles	4 days separation from partner	Long-term intracerebroventricular infusion of a non-selective corticotropin-releasing factor (CRF) receptor antagonist.	Bosch et al., 2009
Chronic social isolation	Male Wistar rats	21 days	Suppressed proplastic response and promoted proapoptotic signaling in prefrontal cortex, mediated by unbalance in glucocorticoid receptor and NFkB Transcription factors.	Djordjevic et al., 2010
Social isolation Intrahippocampal interleukin-1 receptor antagonist	Adult male Sprague-Dawley rats	6 h after contextual fear conditioning	Hippocampal-dependent memory impairments induced by elevated levels of brain IL-1 could occur via an IL-1 -induced downregulation in hippocampal BDNF.	Barrientos et al., 2003
Social isolation	Sprague-Dawley rats, 2 months old, male	8 weeks	Reduction on BDNF protein concentrations in the hippocampus.	Scaccianoce et al., 2006
Social isolation, oxytocin administration	Prairie voles: female, adult (60–90 days old)	4 weeks	Oxytocin can prevent effects of social isolation.	Grippo et al., 2009
Social isolation in experiment 1	Prairie voles: female/male, adult (60–90 days old)	4 weeks	Elevated plasma oxytocin and oxytocin immunoreactive cell density in females.	Grippo et al., 2007
Social isolation	Male C57BL/6 mice	3 months	Changes in methylation in the midbrain	Siuda et al., 2014
Individual housing	Male Wistar rats	12 weeks	Sympathetic nervous system: immunocompetent tissues are depleted of catecholamine, this leads to an impairment of immune response.	Gavrilovic et al., 2010
Social isolation	Male Wistar rats, 45 days old at start	12 weeks	Increased Neuropeptide Y in caudate putamen, more explorative rats.	Thorsell et al., 2006
Social isolation stress	Mice	2 weeks	-Upregulation of the neuropeptide tachykinin 2 (Tac2)/neurokinin B (NkB). -Nk3R antagonist prevented the effects of SI.	Zelikowsky et al., 2018
Social isolation Breast cancer	Mouse model of “triple-negative” breast cancer	12 weeks	Increase in mammary tumor growth and metabolic gene expression.	Volden et al., 2013
Social isolation	Female C3 (1)/SV40 T-antigen mice	9.5 weeks	Significantly larger mammary gland tumors burden and increased expression of key metabolic genes.	Williams et al., 2009
Social isolation during adolescence	Male Wistar rats	3 weeks	-Social isolation in adulthood: reduced systolic arterial pressure and increased diastolic arterial pressure. -Most changes caused in adolescence can be reversed by later group housing, except for body weight and baroreflex sensitivity.	Cruz et al., 2016
Social isolation	Prairie voles	4 weeks	Beneficial effects of an enriched environment on depression- and anxiety-relevant behaviors.	Grippo et al., 2014

Prolonged social isolation also affects different aspects of physiology. It can impair neurogenesis in the olfactory bulb (OB), the ventral hippocampus (VH)^,^ and the dentate gyrus (DG), and lead to reduced volume of some of these structures and the prefrontal cortex (Lu et al., 2003; Day-Wilson et al., 2006; Guarnieri et al., 2020). The loss of medial prefrontal cortex volume, but not its total number of neurons, resembles that observed in individuals with schizophrenia (Day-Wilson et al., 2006). Social isolation also affects the activity of the hypothalamic-pituitary-adrenal (HPA) axis, which controls the reaction to stress. In prairie voles, chronic isolation differentially affects the expression of the corticotropin-releasing factor receptor 2 (*CRF2)* between the hippocampus and the hypothalamus (Pournajafi-Nazarloo et al., 2011), two brain regions with major roles in regulating stress responses.

Notably, social isolation can promote tumor progression in animal models (Williams et al., 2009; Volden et al., 2013), and correlats with increased expression of key metabolic genes, upregulated lipid synthesis, and glucose metabolism in pre-malignant mammary gland (Williams et al., 2009) and mammary adipocytes (Volden et al., 2013).

### Molecular Underpinnings of Social Isolation

#### Social Isolation and Loneliness Can Be Influenced by Genetic Variation

Loneliness is a social state strongly associated with mortality that is influenced by genetic variation (Gao et al., 2017; Day et al., 2018). A genome-wide association study (GWAS) including almost half a million participants from the UK Biobank study revealed the existence of genetic variants associated with loneliness and regular participation in social activities (Day et al., 2018). A total of 15 genomic loci were significantly associated with loneliness. Interestingly, the association was stronger in regions close to genes expressed preferentially in the brain where they are enriched for epigenetic modifications (Day et al., 2018), suggesting that loneliness can be influenced by genetic variants affecting the activity of regulatory elements in the brain. Interestingly, the expression of 8 genes was linked to susceptibility to loneliness: *GPX1, C1QTNF4, C17orf58, MTCH2, BPTF, RP11-159N11.4, CRHR1-IT1*, and *PLEKHM1*. The case of *BPTF* is of interest as it encodes the Bromodomain PHD finger transcription factor (BPTF) which is the largest subunit of the nucleosome remodeling factor (NURF), a major regulator of chromatin structure and gene expression (Barak et al., 2003; Stankiewicz et al., 2017). *BPTF* is highly expressed in the fetal brain and the brain of patients with neurodegenerative conditions, such as Alzheimer’s disease (Bowser et al., 1995). Mutations in *BPTF* have been found in patients with intellectual disability, speech delay, and microcephaly, while genetic inactivation of BPTF in Zebrafish leads to neurodevelopmental phenotypes (Stankiewicz et al., 2017). Therefore, genetic variation affecting the expression of *BPTF* could influence neurodevelopment and social states such as loneliness. Overall, results derived from GWAS suggest that in addition to life experiences, a specific genetic composition could influence social isolation and social interaction. However, whether these genetic associations truly influence brain development and function remains to be determined. Modeling genetic variants identified in humans using murine models and CRISPR-Cas9 editing (Zhu et al., 2019; Sandoval et al., 2020) could prove valuable to decipher the functionality of genetic variants associated with loneliness. Furthermore, it would be of great interest to increase the population diversity of GWAS to provide a comprehensive catalog of genetic variations influencing social behavior across human populations.

#### Social Isolation Induces Changes in Gene Expression

Conversely to the observation that loneliness is influenced by genetic makeup, social experiences can themselves alter gene transcription and have consequences for behavioral responses. In particular, social isolation can modulate gene expression across tissues in many species, from *Drosophila* to mammals (Wallace et al., 2009; Zelikowsky et al., 2018; Agrawal et al., 2020). In *Drosophila*, adult male flies exposed to social isolation for 4 days show robust changes in the expression of 90 genes mostly related to immune response (Agrawal et al., 2020). This is consistent with findings that social isolation modulates immune responses and induces inflammation (Powell et al., 2013; Cole et al., 2015), a condition also associated with depressive-like behaviors in animal models and depression in humans (Ma et al., 2020). The brain-specific neuropeptide *Drosulfakinin* (*Dsk*) was shown to be upregulated in the head of socially isolated males. It was proposed to act as a brake for aggressiveness induced by social isolation as *Dsk* knockdown increases aggressive behaviors of isolated male flies (Agrawal et al., 2020). Notably, its mammalian homolog cholecystokinin (CCK) regulates aggression and anxiety and has been implicated in panic disorder (Zwanzger et al., 2012; Katsouni et al., 2013). CCK transcription can also be modulated by other stressors such as maternal separation (Weidner et al., 2019).

In rodents, chronic social isolation stress can trigger widespread changes in the transcription of protein-coding and non-coding genes (Karelina et al., 2009; Wallace et al., 2009; Liu et al., 2012a; Jin et al., 2016; Kumari et al., 2016; Verma et al., 2016, 2018; Zelikowsky et al., 2018; Mavrikaki et al., 2019; Chang et al., 2020). In adult mice, social isolation for 8 weeks induces transcriptional changes in the myelin genes *Mbop* and *Mobp* in oligodendrocytes of the prefrontal cortex (PFC) (Liu et al., 2012a). Two weeks of social isolation induces a gradual transcription of *Tact2* gene in the brain and peripheral endocrine tissues such as testis (Zelikowsky et al., 2018). *Tact2* codes for the neuropeptide neurokinin B (NkB), necessary for behavioral responses observed in mice subjected to chronic social isolation (Zelikowsky et al., 2018). In rats, prolonged social isolation for 6–12 weeks induces changes in gene expression in the cortex and the nucleus accumbens shell (NAcSh), a brain region important for responses to emotional stimuli (Wallace et al., 2009; Kumari et al., 2016). In cortex, post-weaning social isolation increases the expression of the brain-derived neurotrophic factor (BDNF), the cAMP response element binding protein (CREB-1), and the histone acetyltransferase CREB-1 binding protein (CBP), but reduces the transcription of the histone deacetylase-2 (HDAC2). In the NAcSH, adult chronic social isolation also upregulates many genes coding for K^+^ channels and major regulatory proteins such as the activating transcription factor-2 (ATF2), Janus kinase and genes coding for epigenetic factors such as the histone deacetylase-4 (HDAC4) (Wallace et al., 2009). This suggests that chronic social isolation can potentially rewire gene regulatory networks by altering the amount of activity-dependent transcription factors and chromatin-modifying proteins.

Social isolation in rodents can also affect the expression of non-coding RNAs like miRNAs (Kumari et al., 2016; Verma et al., 2018; Mavrikaki et al., 2019; Antony et al., 2020, p. 181; Chang et al., 2020). Prolonged isolation of postnatal rats resulted in differential miRNAs expression in the anterodorsal bed nucleus of the stria terminalis (adBNS), a region involved in anxiety responses (Mavrikaki et al., 2019). A total of 12 miRNAs were differentially regulated in both socially-isolated males and females, with the majority being downregulated, e.g., miR-181c, miR-143, miR-29a, miR-434, and miR-22 (Mavrikaki et al., 2019). Interestingly, the level of miR-29a was also altered in other tissues such as the oral cavity (Yang et al., 2013), suggesting systemic responses to social isolation. miR-181c expression was also downregulated in the brain of isolated mice after stroke (Verma et al., 2018; Antony et al., 2020) while the levels of miR-181a are affected in blood of adult humans with a history of childhood trauma (Mavrikaki et al., 2019), suggesting that miR-181 family members could be a common target of stress responses in mammals.

Changes in miRNAs expression after social isolation can vary depending on sex (Kumari et al., 2016; Mavrikaki et al., 2019). For example, chronic social isolation upregulates miR-132, a direct target of CREB-1, and downregulates miR-134 in the cortex of female rats (Kumari et al., 2016). In female adBNS, twice more miRNAs were affected than in their male counterparts (Mavrikaki et al., 2019), and this correlated with an anxiety behavior (Kumari et al., 2016; Mavrikaki et al., 2019). These findings suggest that chronic social isolation can differentially modulate behavior and transcriptional programs depending on sex. While in females, target genes of miRNAs altered by social isolation are involved in drug addiction and MAPK signaling suggesting effects on reward pathways, in males, target genes are involved in GABAergic synapses thus affect inhibitory neurons (Mavrikaki et al., 2019). Consistently, social isolation increases the propensity to self-administer drugs and to develop addictive behaviors (Green et al., 2010). Overall, different lines of research strongly support that social isolation can alter transcriptional programs in the brain affecting both protein-coding and non-coding genes.

#### Transcription Factors and Epigenetic Mechanisms Modulate Behavioral Responses to Social Isolation

The mechanisms linking social isolation with changes in gene expression likely involve different molecular cascades with one of the major consequences being perturbed activity of transcription factors (Wallace et al., 2009; Kumari et al., 2016). In the rodent brain, the activity of the transcription factor CREB is diminished in NAcSh of rats exposed to chronic social isolation (Wallace et al., 2009). CREB has been associated with differential expression of a subset of genes, like those coding for K + channels, in the NAcSh of socially isolated rats. Notably, CREB overexpression is sufficient to revert the anxiety-like behavior observed in isolated individuals but not the anhedonia-like phenotype (Wallace et al., 2009). This suggests that CREB is a major player in the regulation of emotional hyper-reactivity in NAcSH and that additional molecular pathways likely regulate other behavioral abnormalities observed in socially-isolated animals. A major question regarding the role of CREB in social isolation is the molecular nature of its reduced activity during prolonged social isolation. To date, it is unknown whether transcriptional or post-transcriptional mechanisms operating in the NAcSh are responsible for its reduced regulatory activity during chronic social isolation.

Classical epigenetic mechanisms for the control of gene expression are also implicated in the effects of prolonged social isolation (Weaver et al., 2004; Murgatroyd et al., 2009; Gapp et al., 2014; Siuda et al., 2014; Wang et al., 2017). Intermittent social isolation in early postnatal life in rodents, such as induced by maternal separation, can modulate DNA methylation and histone post-translational modifications at regulatory elements of genes involved in stress reactivity including the glucocorticoid receptor (GR) gene (*Nr3c1*) (Weaver et al., 2004) and the mineralocorticoid receptor (MR) gene (*Nr3c2*) (Gapp et al., 2014). This has been associated with a rewiring of stress responses and behavioral adaptation. Chronic social isolation during the juvenile period can also alter the epigenome. Pups at postnatal day (PND) 21 subjected to social isolation for 2 months show a global increase in the level of the repressive histone post-translational modification H3K9me2 in neurons, an effect correlated with increased transcription of the H3K9me2 histone methyltransferase (HMT) G9a and GLP in the hippocampus (Wang et al., 2017). In adult male mice, chronic social isolation for 3 months induced a significant global increase in DNA methylation, H3K4 di, and trimethylation as well as a trend toward an increase in the global levels for H3K9ac, in the midbrain (Siuda et al., 2014). In all cases, the increase in epigenetic modifications was associated with an increase in the catalytic processes leading to such epigenetic modifications. For example, H3K4 HMT activity was significantly enhanced in the midbrain of socially isolated male mice, which could suggest increased transcription of genes coding for H3K4 HMT (Siuda et al., 2014). Prolonged social isolation also increased the transcription of genes coding for HDACs such as *Hdac1* and *Hdac3* which correlated with decreased CpG methylation at their promoter regions (Siuda et al., 2014), supporting the hypothesis that chronic isolation can perturb gene regulatory networks by altering epigenetic modifiers. In contrast, the transcription of the gene coding for the serotonin transporter *Slc6a4* was markedly reduced by social isolation and this correlated with increased DNA methylation at its promoter region (Siuda et al., 2014).

### Non-coding RNAs Are Major Regulators of Social Behavior

Non-coding RNAs such as miRNAs and lncRNAs are major regulators of gene expression across the animal kingdom (Jonas and Izaurralde, 2015; Engreitz et al., 2016; Kim et al., 2016; Li and Fu, 2019). Although different lines of evidence suggest that ncRNAs are transcriptionally altered in the brain of rodents after social isolation, direct and functional evidence on their contribution to behavioral and physiological consequences of prolonged social isolation is still sparse (Verma et al., 2018; Antony et al., 2020; Chang et al., 2020). However miRNAs and lncRNAs were proven to modulate social behaviors which are also altered as the result of prolonged social isolation (Haramati et al., 2011; Dias et al., 2014; Issler et al., 2014; Jin et al., 2016; Zhu et al., 2017; Cheng et al., 2018; Lackinger et al., 2019; Labonté et al., 2020; Ma et al., 2020).

#### MiRNAs

Different miRNAs have been documented to modulate aggressive-, anxiety-, and depression-like behaviors as responses to prolonged social isolation. For example, miR-206 is responsible for the stress-induced aggressive behavior of socially isolated mice via direct targeting of *BDNF* mRNA in the ventral hippocampus (Chang et al., 2020). MiR-34c is downregulated in the brain of socially isolated female rats (Mavrikaki et al., 2019) and it is responsive to chronic stress in the adult central nucleus of the amygdala (CeA) where it has been shown to have an anxiolytic effect when overexpressed (Haramati et al., 2011). Since prolonged social isolation is a form of chronic stress and anxiety a behavioral response of socially isolated female rodents (Kumari et al., 2016; Mavrikaki et al., 2019), miR-34c could be a modulator of anxiolytic responses due to prolonged social isolation.

MiR-135 can modulate serotonin functions by targeting the serotonin transporter *Slc6a4* (Issler et al., 2014), which is downregulated in the midbrain of socially isolated adult mice (Siuda et al., 2014). Consistently, deletion of miR-135 gene in serotonergic neurons results in anxiety- and depression-like behaviors while miR-135 overexpression induces resilience to the behavioral effects of chronic social stress (Issler et al., 2014). The miRNA cluster miR-17-92 is of particular interest as it can also regulate anxiety- and depression-like behaviors by targeting transcripts of the glucocorticoid receptor (GR) pathway in the adult brain (Jin et al., 2016). Deletion of the miRNA cluster in neural progenitors in the adult brain resulted in mice displaying anxiety-, depression-, and anhedonia-like behaviors while miR-17-92 cluster overexpression had anxiolytic and antidepressant-like effects (Jin et al., 2016). Notably, anxiety-, depression-, and anhedonia-like behaviors are all behavioral manifestations of adult rodents exposed to chronic social isolation (Wallace et al., 2009; Zelikowsky et al., 2018) which suggest that chronic pervasive stress can modulate the expression of miRNAs and in such way, impact behavior. In support of this, chronic stress results in reduced expression of the miR-17-92 cluster while the overexpression of the miR-17-92 cluster was anxiolytic and protected against the deleterious effect of chronic stress on neurogenesis (Jin et al., 2016). MiR-137 is another important modulator of social behavior. Heterozygous mice for miR-137 show impaired social behaviors, such as reduced social preference toward other mice, as well as impaired response to social novelty (Cheng et al., 2018), all behavioral manifestations of prolonged social isolation in rodents.

The miR-379-410 cluster is the best-characterized group of miRNAs with a demonstrated role in fine-tuning social behavior in mammals (Lackinger et al., 2019). It is specifically expanded in placental animals and contains 38 miRNAs with documented roles in neuronal processes. Constitutive removal of the entire cluster results in hyper-social behavior characterized by increased ultrasonic vocalizations both during early and juvenile postnatal life, exaggerated reciprocal social interactions, and increased social approach behavior, suggesting that such miRNAs as a group can function to buffer social behavior in mammals (Lackinger et al., 2019). Knockout mice also had reduced repetitive behaviors and attenuated anxiety-related behaviors. Molecularly, the loss of the miR-379-410 cluster leads to a major up-regulation of the transcript levels for more than 3,000 genes in neurons, consistent with the role of miRNAs in suppressing gene expression. Interestingly, some of the up-regulated genes code for glutamate receptor components which was linked with increased neuronal excitability in the hippocampus and hyper-social behavior (Lackinger et al., 2019). Therefore, the miR-379-410 cluster is a genomic regulatory hub for the fine-tuning of social behavior in mammals. Whether members of the cluster are implicated in behavioral abnormalities due to prolonged social isolation remains to be determined.

While it is clear that miRNAs are transcriptionally dysregulated by social isolation and some of them directly modulate behaviors characteristic of chronically isolated animals, manipulating specific miRNAs *in vivo* has recently emerged as a promising therapeutic approach to ameliorate the negative effects of social isolation on behavior and physiology (Verma et al., 2018; Antony et al., 2020; Chang et al., 2020). For example, inhibition of miR-206 in the hippocampus of socially isolated mice or intranasal administration of an antagonist of miR-206 eliminates stress-provoked attacks via BDNF upregulation (Chang et al., 2020). Also, social isolation can negatively influence stroke recovery in humans and rodents and this has been associated with the dysregulation of miRNAs, such as miR-181c and miR-141, in a mouse model of stroke (Verma et al., 2018; Antony et al., 2020). Mice that were socially isolated post-stroke showed a gradual decrease in the levels of miR-181c in the ipsilateral cortex as compared with group-housed mice also subjected to stroke. Remarkably, the systemic upregulation of miR-181c using a miRNA mimic significantly increased miR-181c levels in the brain and improved survival rate after stroke in isolated mice. This also partially rescued locomotor effects and ameliorated anxiety. Molecularly, the re-establishment of miR-181c levels reduced glial activation in isolated mice (Antony et al., 2020), a remarkable finding as glia activation after stroke has been related to increased inflammation and poorer prognosis (Xu et al., 2020). This data suggest that social isolation could compromise neuroinflammatory responses in the brain after stroke. In support of this, social isolation after stroke impairs the transcriptional upregulation of interleukin-6 (IL-6) in the brain (Karelina et al., 2009). While IL-6 is a cytokine involved in the induction of inflammatory responses, IL-6 induction in the brain is neuroprotective (Loddick et al., 1998). Importantly, the systemic inhibition of miR-141c, which is upregulated in the brain of socially isolated mice after stroke, resulted in the transcriptional upregulation of *IL-6* (Verma et al., 2018). Thus, miRNAs act as major modulators of inflammatory responses via regulation of pro-inflammatory genes in the context of social isolation after stroke.

#### LncRNAs

LncRNAs can also affect social behavior in mice through different mechanisms. The antisense lncRNA of synapsin II (AtLAS) is differentially expressed in the mPFC between dominant and subordinate mice (Ma et al., 2020) and its downregulation in excitatory neurons of the mPFC is sufficient to establish social dominance in grouped mice. Since chronically isolated mice have altered behaviors toward other individuals, such as increased aggression but also blunted response to social novelty (Zelikowsky et al., 2018), it is possible that lncRNAs are important modulators of behavioral responses due to chronic social isolation in mammals. Consistently, aggressive behaviors have also been associated with changes in lncRNAs expression (Punzi et al., 2019; Labonté et al., 2020). The monoamine oxidase A (MAOA) associated ncRNA (MAALIN) is a lncRNA located in the 3′ intergenic region separating the tail-to-tail oriented MAO genes A and B. In humans, the promoter of MAALIN is hypomethylated in neurons of the DG from suicidal subjects with a history of impulsive aggressive disorders and this correlates with lower expression of *MAOA* gene, which has been implicated in aggressive disorders both in humans and animals (Labonté et al., 2020). Overexpression of MAALIN in the hippocampus of aggressive mice induces a discrete downregulation of *MAOA* and results in a trend for increased duration of attacks toward other mice, suggesting that MAALIN could affect aggressive behavior (Labonté et al., 2020), a stereotypic behavioral response for male mice exposed to chronic social isolation.

### Neuropeptides Are Major Drivers of Behavioral Responses to Social Isolation

Neuropeptides are major modulators of the behavioral effects observed during extended periods of social isolation across the animal kingdom. In *Drosophila*, the neuropeptides drosulfakinin and tachykinin modulate aggressive behavior in isolated male flies (Asahina et al., 2014; Agrawal et al., 2020). In mice, the expression of the neuropeptide NkB is sufficient and necessary for the behavioral abnormalities observed in socially isolated mice (Zelikowsky et al., 2018). Interestingly, NkB acts regionally in different brain areas to modulate specific behavioral responses due to chronic social isolation stress (Zelikowsky et al., 2018). Therefore, neuropeptides in conjunction with activity-dependent transcription factors, epigenetic modifiers, and ncRNAs are major modulators of behavioral and physiological responses to social isolation.

### Behavioral Implications of Quarantine During Epidemics: A Cautionary Tale

In the past centuries, the timely implementation of isolation and quarantine of human populations has shown to be an effective public health intervention to stop the spread of viruses, such as the Ebola virus, MERS-CoV, SARS-Cov, and more recently, SARS-CoV2, the causal agent of COVID-19 (Hull, 2005; Pellecchia et al., 2015; Yoon et al., 2016; Prem et al., 2020). Countries all over the world have applied this strategy, resulting in mandatory or voluntary confinement for several months for more than a third of the world’s population up to this point.

Various countries pursued different approaches to prevent and reduce the spread of the virus. The first and strictest type of quarantine was enforced in Wuhan, China, the origin of the coronavirus outbreak (Prem et al., 2020). In some areas of the city, residents were completely forbidden to leave their home. Authorities went from door to door for health checks and forced the ill into isolation (Wuhan’s coronavirus outbreak: life inside the quarantine). Italy was the second country to enforce quarantine, then most European countries followed with different level of restrictions. Some countries pursued a more relaxed approach, such as Sweden where confinement was not mandatory, resulting in differences on the overall infectious and death rate due to COVID-19 (Habib, 2020).

While isolation refers to the separation of infected people from those who are healthy, quarantine separates and restricts the movement of people who might be infected but are not yet symptomatic. Physical distancing reduces the frequency and closeness of social contact between people. Although quarantine has been successful in slowing down the spread of the virus, poor implementation can cause additional problems in the exposed people (Pellecchia et al., 2015; Brooks et al., 2020; Buttell and Ferreira, 2020). The current quarantine due to COVID-19 has increased domestic violence, fear of people losing their jobs, reduced physical activity, altered sleep, and increased anxiety (KANTAR, 2020; How the Pandemic Could Be Messing With Your Sleep; Agren et al., 2020; Bouillon-Minois et al., 2020; Economic Commission for Latin America and the Caribbean, 2020; Mahase, 2020; Mazza et al., 2020; Spinelli et al., 2020; Thomas et al., 2020). These effects can even be more pronounced in people in developing countries where most of the population lives under the poverty line, including nations in Africa, Asia, and Latin America. Residents of such countries are in a tremendous hazard on suffering from lasting effects of forced confinement as they can not fulfill even their most basic need. They experience the quarantine as a major physical and psychological stressor for extended periods of time (Madhav et al., 2017; Yatham et al., 2018; Agren et al., 2020; Economic Commission for Latin America and the Caribbean, 2020).

More than 50 years of research in animal models and humans have conclusively shown the detrimental effects of chronic stress on health, highlighting the necessity for more empathic interventions to protect or reduce the sequelae of confinement on mental health and well-being of the population. Simple yet effective strategies could be implemented to reduce social isolation and perceived loneliness among older people, which are a sector of the population at risk to experience the detrimental effects of social isolation (Gardiner et al., 2018). Animal interventions, like animal-assisted therapy or having a pet has been shown to alleviate loneliness in the elderly (Shankar et al., 2011; Krause-Parello, 2012). The use of electronic devices, specifically computer and internet in older adults has also been found to decrease loneliness (Heo et al., 2015). In this regard, the use of a mobile phone for sociability has been associated with decreased loneliness, particularly when used in the context of face-to-face interactions (Wang et al., 2018). Therefore, the knowledge gained by previous research on the biological effects of social isolation on behavior has been an important driving factor for the realization that quarantine can have long-lasting effects on the population.

## Conclusion

Prolonged social isolation has detrimental effects on humans and animals. In humans, chronic social isolation perturbs physical and mental health and we are just starting to uncover the molecular mechanisms driving behavioral effects associated with social withdrawal. Evidence derived from different animal models strongly suggests that social isolation can induce transcriptional changes in different brain areas fundamental for memory and cognition and also relevant for the modulation of mood and even addictive behaviors. Some of the affected genes are major transcriptional regulators such as the AP-1 transcription factors and CREB, both mediators of transcriptional responses due to neuronal activation in mammals (Yap and Greenberg, 2018). Furthermore, important epigenetic modifiers such as the H3K9me2 histone methyltransferase G9a and histone deacetylases like HDAC-2 and -4, as well as regulatory ncRNAs like miRNAs are also dysregulated, suggesting that social isolation could remodel chromatin and impact steady-state or stimulus-dependent transcriptional responses. While current findings suggest such a possibility, direct causal evidence linking the potential mediators, e.g., transcription factors and epigenetic modulators, with the establishment and maintenance of behavioral and physiological abnormalities associated with social isolation, is still sparse. A major missing information also is the identification of signaling pathways responsible for transcriptional events observed in the brain of socially isolated animals such as CREB activation or transcriptional downregulation of HDACs. Also, the molecular events leading to specific regulation of a subset of miRNAs that modulate important signaling molecules such as BNDF and IL-6 during social isolation are not known.

Furthermore, although available evidence suggests that GR signaling is implicated in the response to acute social isolation in mice (Kamal et al., 2014), whether it contributes to transcriptional effects observed during chronic social isolation is unknown. Based on the available evidence, we envision that chronic social isolation induces remodeling of chromatin structure and organization as a consequence of exposure to chronic stress. Such modification could affect not just brain cells but also other tissues, persistently modifying regulatory programs which in turn change behavior and physiology.

From a public health perspective, major attention should be paid to the physiological and psychological consequences of social withdrawal on the general population. Given that loneliness in humans has been documented to be linked to all-cause increased mortality and with an effect on mortality comparable to smoking, it is fundamental to gain better knowledge of the molecular mechanisms that promote the behavioral and physiological effects of isolation with the long-term goal to develop new pharmacological and non-pharmacological interventions. While in most cases, social isolation has detrimental effects on the exposed individual in humans and animals, it is possible that some individuals show some resilience. It may be linked to better coping strategies, a isolation habituated state due to a lifestyle based on loneliness, or a natural lower sensitivity to such social stress.

Finally, current actions to mitigate the pandemic of COVID-19 is a call to revisit and implement the best possible public health interventions to protect people against infectious diseases without affecting their physical and mental health. The imposed regulations by governments around the world may have consequences that people do not anticipate and may reverberate for years and possibly decades. Given that the emergence and spread of viruses that infect humans are and will be a constant thread for humankind, a more thoughtful strategy is needed to reduce social interaction while taking into consideration the extraordinary impact that social interactions can have in life.

## Author Contributions

AJ, ZL, and VS wrote a draft of the review, and RGA-M and IMM finished it.

## Conflict of Interest

The authors declare that the research was conducted in the absence of any commercial or financial relationships that could be construed as a potential conflict of interest.
